# Cough Audio Recognition for Early Detection of Respiratory Diseases: Algorithm Development and Validation Study

**DOI:** 10.2196/77295

**Published:** 2026-05-07

**Authors:** Wensheng Sun, Jiahao Zou, Na Yin, Wenying Fang, Jimin Sun, Ziping Miao, Shigui Yang

**Affiliations:** 1 School of Communication Engineering, Hangzhou Dianzi University, Hangzhou, China; 2 Chaohu Hospital affiliated with Anhui Medical University, Chaohu, China; 3 Zhejiang Key Lab of Vaccine, Infectious Disease Prevention and Control, Zhejiang Provincial Center for Disease Control and Prevention, 3399 Binsheng Rd, Binjiang District, Hangzhou, 310051, China, 86 057187115138; 4 Department of Emergency Medicine, The Key Laboratory of Intelligent Preventive Medicine of Zhejiang Province, Second Affiliated Hospital Zhejiang University School of Medicine, Hangzhou, China

**Keywords:** cough classification, associated diseases, ResNet18, deep learning, attention mechanism

## Abstract

**Background:**

Coughing is a common clinical symptom and a protective respiratory reflex closely associated with various respiratory system diseases. The acoustic characteristics of cough sounds are influenced by underlying pathological factors, with distinct acoustic signatures corresponding to different etiologies. Through rigorous analysis of these sounds, rapid identification and preliminary diagnosis of related conditions may be achieved. This approach holds great potential for broad application in mobile health and ubiquitous health platforms.

**Objective:**

This study aimed to explore the application of acoustic analysis of cough sounds in the diagnosis of respiratory diseases to enhance the diagnostic efficiency of health care professionals.

**Methods:**

In this study, we conducted extensive data collection, including voluntary cough audio recordings from patients diagnosed with respiratory diseases (eg, chronic obstructive pulmonary disease, lung cancer, COVID-19, and pneumonia) and from healthy participants. A total of 2610 audio samples were collected. We incorporated a channel attention mechanism (CAM) into the final convolutional block of each residual block in the ResNet18 neural network, thereby constructing the CAM-ResNet18 neural network model. The recorded cough audio samples were converted into spectrograms to form the input dataset for model training. The CAM-ResNet18 model was trained on the training set of this dataset, with iterative parameter adjustments until convergence was achieved. Finally, spectrograms from the test set were fed into the pretrained model for accurate classification of the cough-related conditions.

**Results:**

Experimental results on the collected audio dataset demonstrate that the proposed CAM-ResNet18 model achieves an accuracy of 83.9% and an average *F*_1_-score of 82.52% in classifying 5 types of cough sounds. In comparison, the traditional ResNet18 model achieves an accuracy of 78.16% and an average *F*_1_-score of 78.29%, indicating a clear performance improvement with the integration of the CAM.

**Conclusions:**

The experimental results validate the effectiveness of the proposed method, highlighting its significant potential for application in clinical diagnosis.

## Introduction

Coughing is one of the common clinical symptoms, often serving as a prominent symptom in various diseases such as chronic obstructive pulmonary disease (COPD), lung cancer, COVID-19, and pneumonia [1,2]. Accurately discerning the association between cough and these related diseases is of paramount importance for clinical diagnosis and treatment. However, conventional methods for cough diagnosis frequently rely on the expertise and observation of medical professionals, posing issues related to subjectivity and uncertainty [3,4].

With the continuous advancement of machine learning technology, the ability to use computer-driven analysis and determination of cough audio data has become feasible, offering a novel avenue for the diagnosis of cough-related diseases [5-7].

Most of the current cough detection and classification methods use standard speech recognition algorithms to extract feature parameters from cough audio [8]. The most common methods in this regard include short-time energy, amplitude, average zero-crossing rate in the time domain, and linear prediction cepstral coefficients (LPCCs) [9], perceptual linear prediction (PLP) [10], and Mel-frequency cepstral coefficients (MFCCs) in the frequency domain [11-13]. These traditional methods provide effective means for extracting features from cough sounds and have played a significant role in early cough classification research. With the advancement of machine learning technology, significant progress has been made in cough detection and classification. People can now use artificial neural networks to recognize and classify cough sounds, achieving a higher level of accuracy. Deep learning models, such as a convolutional neural network, residual neural network, long short-term memory network, and so on, have demonstrated excellent performance in cough sound classification tasks. Many studies provide novel and effective methods that demonstrate high performance and practical value in clinical practice.

The focus of Liyue et al’s [14] research is on classifying pneumonia and asthma based on children’s cough sounds. Initially, cough sound segments from patients with asthma and pneumonia undergo preprocessing steps, such as pre-emphasizing and framing. Subsequently, a feature extraction process is used, which includes the extraction of 24-dimensional MFCCs and short-term energy mixed feature parameters. Classification is carried out using support vector machines [15,16], achieving a sensitivity of 96.3% and a specificity of 93.6%, demonstrating high feasibility and effectiveness. However, the limited sample size used in their experiments may restrict the generalizability of their model to broader datasets and the reliability of practical applications.

Huang et al [17] conducted a critically important study in the field of cough detection and classification. Their research primarily focused on using spectrogram-based methods in conjunction with a parallel 1D deep convolutional neural network (CNN) to extract new features for the classification of dry coughs and wet coughs. They performed feature analysis using linear predictive coding coefficients, Mel spectrograms, MFCCs, and other methods. They extracted the first and second derivatives of the original spectrogram and created a single feature vector. This feature vector was combined with a parallel 1D deep convolutional network. The validation on the dataset indicated that the performance of the parallel flow network significantly surpassed that of the single flow network, achieving an *F*_1_-score of 98.61%. This result underscores the effectiveness of their approach. However, this method used various manually designed feature extraction techniques, which require manual selection and design, and may not fully capture all complex variations in cough sounds.

In their research on cough detection and classification systems, Bales et al [18] primarily focused on using CNNs for feature extraction from cough sounds. They used a low-complexity network structure and had a limited dataset. Nevertheless, their developed cough detection and classification system successfully identified three respiratory diseases: bronchitis, bronchiolitis, and whooping cough. The success of this system serves as evidence of the significant potential of artificial neural networks in the task of cough sound detection and classification.

In their study on the task of patient with COVID-19 detection, Bansal et al [19] proposed an audio classifier based on a CNN. They used an open dataset that had been manually labeled into COVID and non-COVID classes. Extracted MFCC features were used as input and fed into the CNN for training and classification. Through testing and validation of the data, the MFCC-based method achieved an accuracy of 70.6% and a sensitivity of 81%, demonstrating its effectiveness in identifying patients with COVID-19. However, in clinical practice, the complexity and computational costs of their method may limit its application in mobile health settings.

This study aimed to explore a machine learning–based method for cough audio classification and recognition, enabling more precise automated assessment of the relationship between coughing and associated diseases [9,20]. In the context of early disease diagnosis and treatment, this approach has the potential to provide medical professionals with a more accurate and efficient diagnostic tool, facilitating early disease detection and intervention, thereby significantly improving patient treatment outcomes and quality of life [2].

The main contributions of this study are as follows:

1. Proposing the CAM-ResNet18 neural network model, which enhances the model’s ability to focus on key features by introducing the CAM mechanism in the last convolutional layer of each residual block.
2. Converting cough audio into spectrograms, visualizing the spectral features of the audio signals to allow the deep learning model to understand the audio data more intuitively.
3. Constructing a unique large-scale audio dataset by collecting cough audio data from professional hospitals, covering patients with COPD, lung cancer, pneumonia, COVID-19, and healthy participants, providing a solid foundation for model training and evaluation.
4. Demonstrating that the proposed model outperforms traditional models in terms of accuracy and average *F*_1_-score on the collected dataset, validating the effectiveness of the model.

In this research, we selected the ResNet18 residual neural network as the foundational CNN [21,22]. This network has demonstrated outstanding performance in image classification tasks and has found widespread applications in the field of computer vision [22]. To enhance the model’s ability to focus on key features within cough audio, we introduced a channel attention mechanism (CAM). Attention mechanisms have been extensively studied in visual and speech processing tasks and have shown significant performance improvements. They enable the model to concentrate its attention on critical features, leading to more accurate classification [23,24]. To meet the input requirements of the machine learning model, we converted the collected cough audio recordings into spectrograms for representation. Spectrograms offer notable advantages in extracting spectral information from audio signals, aiding machine learning models in comprehending audio data more comprehensively [25]. These generated spectrograms served as input data for the model, leveraging the ResNet18 architecture and incorporating the attention mechanism to enhance the classification and recognition performance of cough audio [26]. This series of processing steps contributes to improving the model’s accuracy and robustness, enabling it to effectively handle the analysis and classification tasks associated with cough audio data. During the research experimental phase, we actively collected a large number of cough audio samples. These samples were obtained from various groups, including patients with different respiratory diseases, such as COPD, lung cancer, pneumonia, and COVID-19, as well as samples from healthy participants, totaling 5 categories. Subsequently, rigorous data preprocessing and feature extraction were performed to ensure data quality and usability. On the constructed dataset, a series of experiments was conducted to assess the effectiveness of the proposed method, with comprehensive comparisons made against traditional classification approaches. The experimental results unequivocally demonstrate a significant advantage of our model in analyzing the association between cough audio and related diseases. This pivotal discovery holds potential clinical application value, presenting a valuable breakthrough in the field of disease diagnosis [27,28].

## Methods

### Ethical Considerations

The investigation received approval from the ethics committee of Zhejiang Provincial Centre for Disease Control and Prevention (2020‐024). Informed consent was obtained from all participants and their legal guardians. Research involving human participants was conducted in accordance with the Declaration of Helsinki. All cough audio data involved in this study have been anonymized and de-identified, with all personal identifiers such as name, gender, age, and medical record number removed. No information that can directly or indirectly identify participants is included, and the study strictly complies with relevant privacy protection regulations and data security requirements for medical research. The research protocol and data collection procedures are in accordance with ethical review requirements, and the privacy and data confidentiality rights of participants are fully protected. As this study is basic algorithmic research without clinical intervention, no financial or material compensation was provided to participants.

### Cough Audio Sample Collection

The collected cough audio data primarily come from several top-tier hospitals in Zhejiang and Anhui provinces. With the explicit consent of the patients, professional physicians were responsible for collecting cough audio data from 5 different categories, including healthy participants, and participants with COPD, lung cancer, pneumonia, and COVID-19. The data collection process took place in a quiet environment, with physicians using standardized recording equipment directed at the source of the patient’s sound to ensure recording quality was not affected by environmental noise. Patients aged between 10 and 90 years were selected from different occupational groups, socioeconomic statuses, and genders. The selection criteria included patients with clear diagnoses and significant cough symptoms, whereas the exclusion criteria included patients unwilling to provide audio data or unable to produce clear cough recordings. The participant population included 724 women and 736 men. Each collected cough audio file was clipped into 3-second audio segments, and each file was labeled with corresponding information, such as the patient’s disease diagnosis, age, and gender. Ultimately, we constructed an audio dataset consisting of 2610 audio samples. To support effective model training and evaluation, this study used 5-fold cross-validation for all experiments. This method effectively avoids inconsistencies that may arise from data splitting biases, further enhancing the reliability of the experimental results. Table 1 illustrates the distribution of these 5 types of cough audio in the dataset.

**Table 1.** The distribution of 5 types of cough audio in the training and testing datasets.

Characteristics	Training, n				
	COPD^a^ (n=420)	Lung cancer (n=435)	Pneumonia (n=413)	COVID-19 (n=398)	Healthy (n=425)
Gender					
Male	317	236	251	252	254
Female	208	309	264	244	275
Age (y)					
10‐14	0	0	23	43	19
15‐24	22	4	23	18	31
25‐34	38	41	60	36	83
35‐44	65	66	57	65	41
45‐54	100	110	81	83	121
55‐64	119	124	95	91	102
65‐74	106	99	86	79	88
75‐90	75	101	91	83	41

a COPD: chronic obstructive pulmonary disease.

### Cough Features

**Figure 1.**
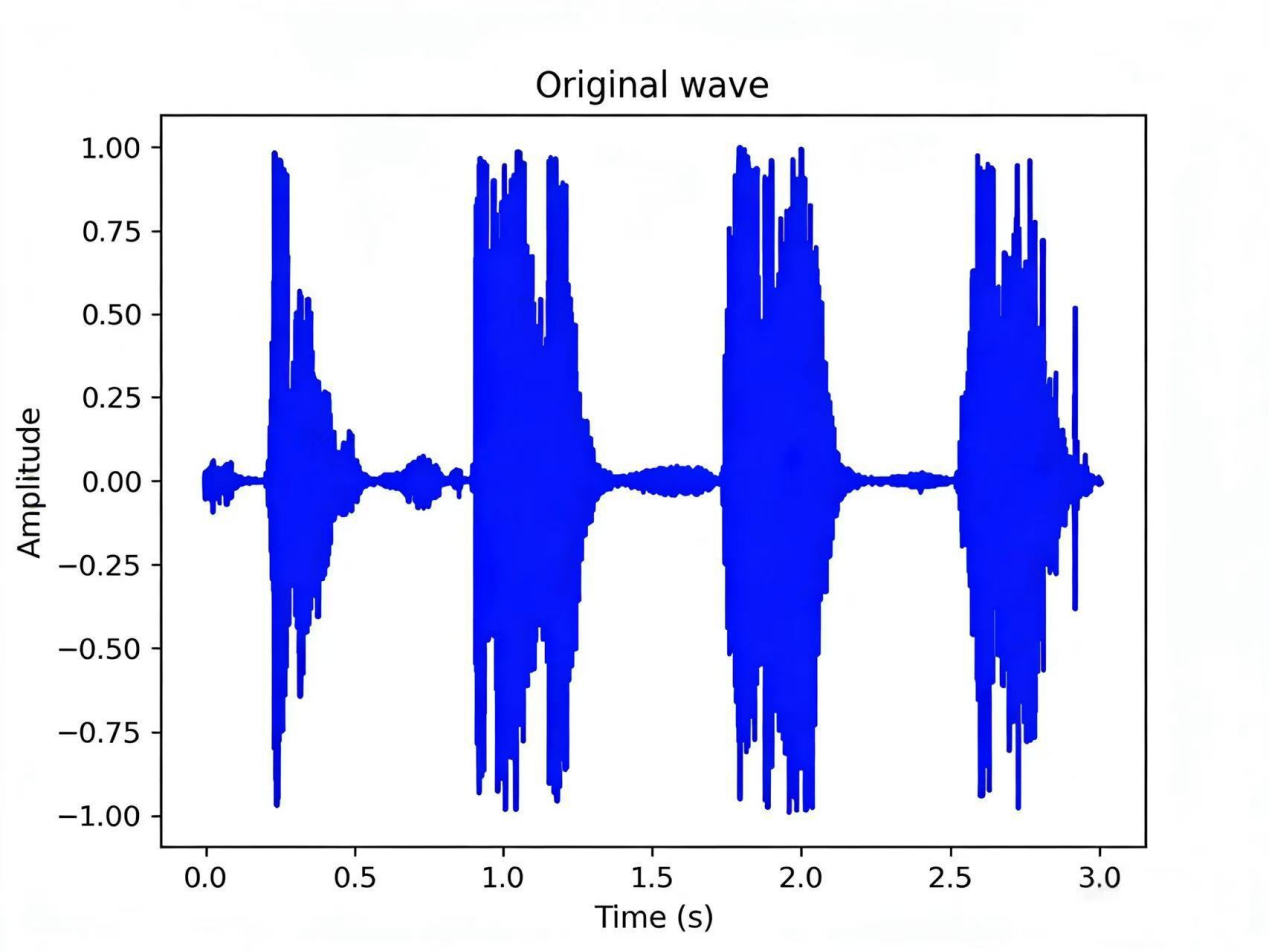
The time-domain waveform of cough audio.

**Figure 2.**
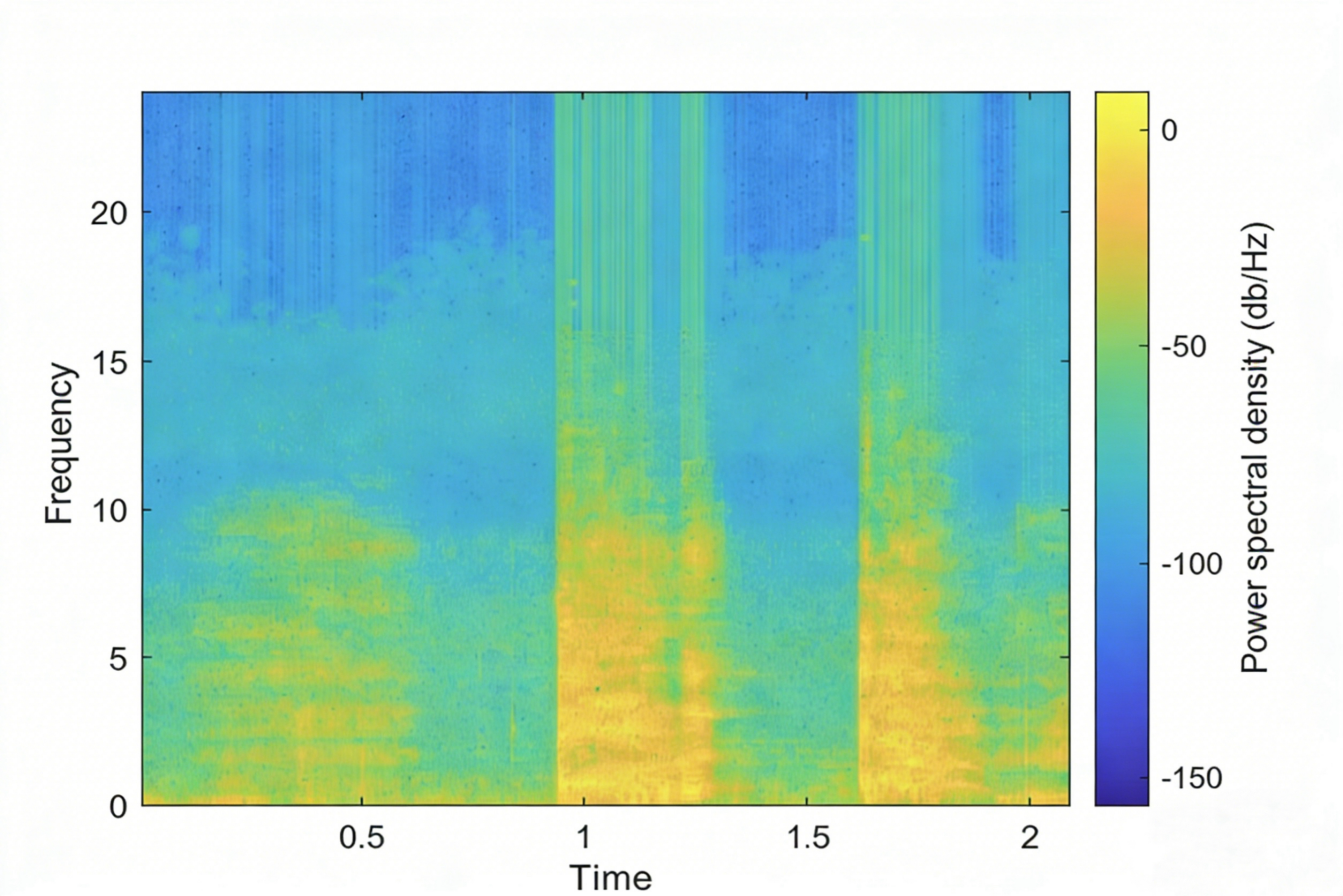
The spectrogram of cough audio.

The general time-domain waveform of cough audio is depicted in Figure 1. When conducting a detailed analysis of cough audio, we typically perform analyses in both the time and frequency domains [29,30]. However, in time-domain analysis, it is impossible to know the distribution of sound at different frequencies, and in frequency-domain analysis, it is impossible to understand how the sound changes over time, and the time-domain and frequency-domain analyses are highly sensitive to noise and signal changes, which will have a great impact on the result analysis. To provide a more intuitive display of the acoustic characteristics of cough audio, we have developed a spectrogram-based representation that combines data points from the spectrogram and time-domain waveform. This approach is used to present rich information related to speech characteristics and facilitates a more comprehensive understanding of the acoustic features of cough audio. The spectrogram representation of cough audio is illustrated in Figure 2.

### Feature Extraction From Cough Audio

Before inputting raw cough audio into the neural network model for the classification of cough-related diseases, a crucial preprocessing step is necessary. This preprocessing procedure consists of 2 fundamental steps: data preprocessing and the generation of spectrograms for the cough audio.

#### Preprocessing

First, preprocessing operations are executed on the raw cough audio, including end point detection and normalization. All cough audio is converted into the WAV format. Background noise segments within the cough audio are manually eliminated through human intervention, and subsequently, all cough audio is segmented into multiple audio segments, each with an approximate duration of 3 seconds. As most of the collected cough audio samples are between 3 and 6 seconds long, selecting a length of 3 seconds helps maintain the relative stability of the audio signal within a short time frame. For audio segments longer than 3 seconds, they are clipped to 3 seconds. For segments shorter than 3 seconds, zero-padding is used to extend them to 3 seconds.

#### Spectrogram

##### Overview

**Figure 3.**
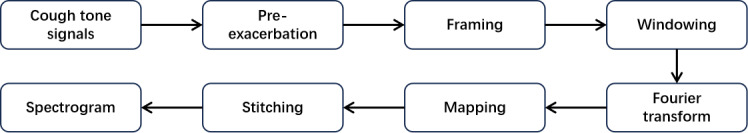
Process flowchart for spectrogram generation.

A spectrogram is a graphical representation used to depict the variation of cough spectra over time. Its horizontal axis represents time, whereas the vertical axis represents frequency. It combines the characteristics of a spectrogram and a time-domain waveform, enabling a clear depiction of how the cough spectrum changes over time. Through spectrograms, phonemic attributes of cough can be better observed, and the identification of cough sounds is improved by observing resonance peaks and transitions. The process of obtaining a spectrogram is illustrated in Figure 3.

Each section’s specific workflow is as follows.

##### Section 1

Pre-emphasis in audio signals is performed to enhance high-frequency components in cough, reducing the influence of lip radiation and improving the high-frequency resolution of the cough signal. Typically, pre-emphasis is achieved using a first-order finite impulse response high-pass digital filter, with a pre-emphasis coefficient typically set to 0.97. Its transfer function is as follows:


(1) $$H\left(z\right)=1-\mu z^{-1}\;\;\;H\left(z\right)=1-\mu z^{-1}\;\;\;0.9\leq \mu \leq 1$$

##### Section 2

Speech signals exhibit short-term stability. After pre-emphasis, these signals are segmented and windowed to divide the original signal into several small blocks, with each block referred to as a frame. Each frame has a length of 25 milliseconds with a frame shift of 10 milliseconds. This is because speech signals can be considered stationary over short periods, and a frame length of 25 milliseconds balances time-domain and frequency-domain characteristics effectively. Once frame segmentation is completed, a window function is applied to each frame to achieve better sidelobe attenuation. Subsequently, a discrete Fourier transform is applied to each frame. Then, each frame undergoes a 512-point fast Fourier transform. Choosing 512 points ensures spectral resolution while also balancing computational efficiency, transforming the time-domain signal into the frequency-domain signal to obtain the necessary spectral information. The discrete Fourier transform formula used is as follows:


(2) $$X\left(k\right)=\sum_{n=0}^{N-1}x\left(n\right)w\left(n\right)e^{-\frac{2\pi i}{N}kn}\;k=0,...,N-1$$

where x(n) represents the speech signal, N is the window size, w(n) is the Hamming window function, and n denotes the time-domain sampling point.

##### Section 3

The process involves stacking the spectral graphs obtained after the discrete Fourier transform for each frame. Subsequently, the amplitudes are mapped to a grayscale representation, where darker colors correspond to higher amplitudes. Finally, by concatenating multiple frames of spectra, a spectrogram is generated.

### CAM Component

**Figure 4.**
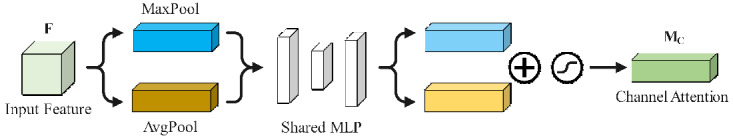
The conceptual flowchart of the channel attention mechanism. MLP: multilayer perceptron.

CAM is an attention mechanism used in image processing. It enhances the expressive power of images by automatically learning the importance of each channel. This helps networks focus on crucial features, suppress unimportant ones, and improve image classification performance and robustness. Figure 4 illustrates the conceptual flowchart of the CAM [31].

The input to CAM is a feature map with dimensions H×W×C, where H represents the height of the feature map, W denotes the width of the feature map, and C is the number of channels. Its fundamental concept is as follows.

First, the input feature map is subjected to both global max-pooling and global average-pooling operations along the spatial dimensions. These operations are performed to compress the spatial dimensions, making it easier to extract the most significant regions in the feature map. The input feature map is processed for global max-pooling and average-pooling based on the following formulas:


(3) $$GlobalMaxPooling\left(x\right)={max}_{i=1}^{h}{max}_{j=1}^{w}x_{i,j}$$

(4) $$GlobalAvgPooling\left(x\right)=\frac{1}{h\times w}\sum_{i=j}^{h}\sum_{j=1}^{w}\;\;x_{i,j}$$

where x is the input feature map, and h and w represent the height and width of the input feature map, respectively.

Second, the results obtained from global max-pooling and average-pooling are fed into a multilayer perceptron (MLP) for feature learning, which involves learning features in the channel dimension and the importance of individual channels. Subsequently, the output from the MLP undergoes addition, followed by applying the Sigmoid activation function to obtain attention weight values for each channel in the input feature map. The final channel attention weights are obtained based on the following formula:


(5) $$\begin{array}{l} M_{c}(F)=\sigma (MLP(AvgPool(F))+MLP(MaxPol(F)))\\ \;\;\;\;=\sigma (W_{1}(W_{0}(F_{avg}^{c}))+W_{1}(W_{0}(F_{max}^{c}))) \end{array}$$

In this context, M_c_(F) represents the channel attention weights, where *F* denotes the input feature map, and c signifies the number of channels. W_0_ and W_1_ represent the parameters learned in the fully connected layers. MLP consists of 2 fully connected layers [32], and σ represents the Sigmoid activation function.

In the CAM module, the ‘shared MLP’ consists of 2 fully connected layers. The first layer has 256 neurons, the second layer has 128 neurons, and the final output is the size of the number of channels, which is used to generate the attention weights for each channel.

Finally, the obtained attention weights are multiplied by the initially input feature map to obtain the feature map weighted by channel attention. This is achieved using the following formula:


(6) $$Z=M_{c}\times \left[\left(F\left(x\right)+x\right)\right]$$

where Z represents the feature map after attention weighting, *M*_*c*_ represents the channel attention weights, x denotes the input, and F(x) signifies the output after passing through convolutional layers and activation functions.

### The Network Architecture of the CAM-ResNet18 Model

**Figure 5.**
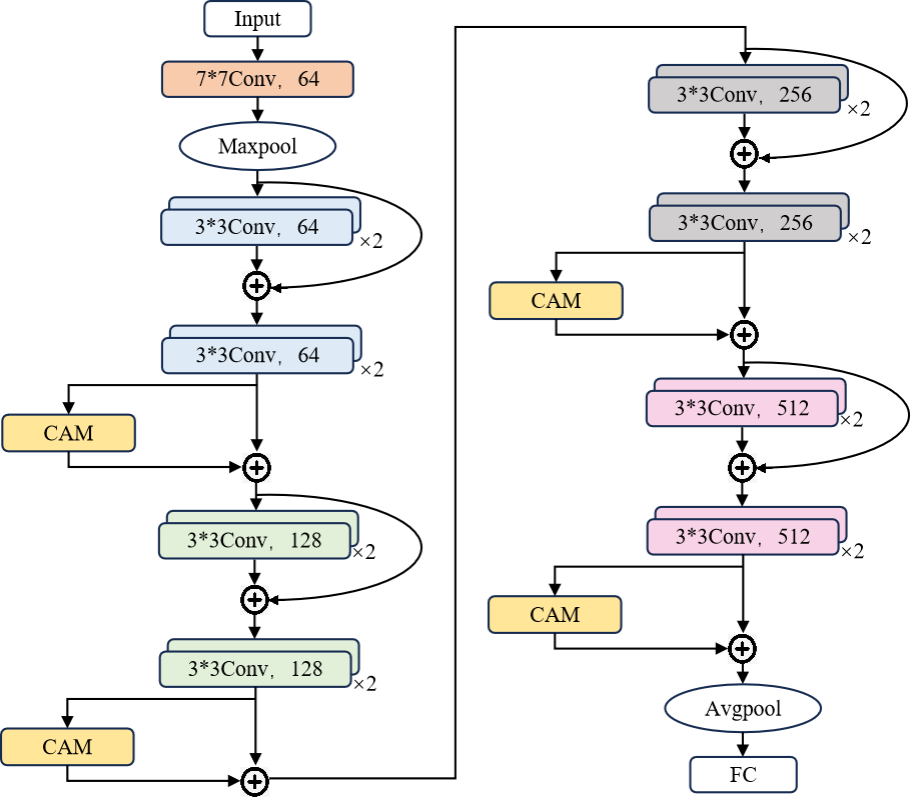
The neural network architecture diagram of CAM-ResNet18. CAM: channel attention mechanism; FC: fully connected.

In this study, the backbone network chosen for the model is the ResNet18 residual neural network. We enhance the neural network model, referred to as CAM-ResNet18, by introducing the CAM. The CAM is added to the last convolutional block in each residual block of the ResNet18. This choice is made because the last convolutional block is the final convolutional operation within each residual block, and as the structure of each residual block in ResNet18 is identical, incorporating the CAM here allows for targeted improvement in the feature learning capability of each residual block. Importantly, it does not affect the feature extraction in other residual blocks, thereby enhancing the model’s ability to distinguish between different types of cough sounds. Figure 5 depicts the neural network architecture of CAM-ResNet18.

The CAM-ResNet18 model network structure consists of the following components:

- Input layer: It receives transformed spectrograms as input, which capture the spectral information of cough sounds.
- Initial convolutional layer and max pooling layer: Initially, the input undergoes a convolutional layer with a kernel size of 7×7, a stride of 2, and padding of 3, resulting in an output of 64 channels. This is followed by a max-pooling operation with a 3×3 kernel size, a stride of 2, and padding of 1, aimed at reducing the feature map’s dimensions.
- Residual blocks: Each residual block consists of two 3×3 convolutional layers and a skip connection, designed to address the issues of gradient vanishing and exploding gradients in deep CNNs.
- CAM is inserted after the last convolutional layer in each residual block, enhancing the learning capabilities of each residual block selectively and improving the model’s focus on critical features. This is accomplished through the following steps:
  - Global max-pooling and average-pooling operations are applied to the feature maps from the final convolutional block. These operations compress spatial dimensions and extract the most salient regions in the feature maps.
  - Fully connected layer: the results obtained after global max-pooling and global average-pooling are fed into an MLP consisting of 2 fully connected layers for feature learning. Subsequently, the Sigmoid activation function is applied to obtain weight values for each channel.
  - Channel weighting: the generated channel weights are applied to the original feature map by multiplying each channel’s features by the corresponding weight, thus achieving channel weighting.

- Global average pooling and fully connected layer: feature maps undergo average pooling to reduce dimensionality and map the features from the final layer to output categories.
- Output layer: feature maps undergo average pooling to reduce dimensionality and map the features from the final layer to 5 output categories, corresponding to the 5 classes.

### Model Training Process

Our model training was conducted with the following environment configuration: Intel Core i5-13600K CPU, 32GB of RAM, CUDA 11.3, the deep learning framework PyTorch 1.12.1, and dated March 3, 2022. We used spectrograms of cough audio from the training dataset as input, which were then fed into the CAM-ResNet18 neural network model for training. During the training process, the hyperparameter settings are presented in Table 2.

**Table 2.** Hyperparameter settings.

Hyperparameter	Value
Loss function	Cross-entropy loss function
Optimizer	Adam
Learning rate	0.001
Training rounds	50
Batch size	8
Weight initialization	He initialization
Learning rate scheduler	StepLR
Step size	7
Attenuation coefficient	0.1

The cross-entropy loss function is used to quantify the disparity between the model’s predicted probability distribution and the actual probability distribution [33,34]. In this experiment, we focus on 5 categories, namely, healthy participants and patients with COPD, lung cancer, pneumonia, and COVID-19. We use one-hot encoding for mathematical representation, where each category is represented as a 5D vector with only 1 dimension set to 1, indicating the presence of that category, while all other dimensions are set to 0.

In the experiment, assuming we input a spectrogram of a lung cancer cough audio, and its true label is represented by a one-hot encoding vector with 5 dimensions [0, 1, 0, 0, 0]. This signifies that only the dimension corresponding to lung cancer has a value of 1, whereas the values on the dimensions corresponding to the other 4 categories are all zero. The model’s output is also a vector with the same dimensions as the true label, where each dimension’s value represents the model’s predicted probability for healthy participants and patients with COPD, lung cancer, pneumonia, and COVID-19. These probability values sum to 1.

To calculate the loss value, we compare the model’s predicted probabilities with the true labels using the following cross-entropy formula:


(7) $$H(p,q)=-\sum_{i=1}^{n}p(x_{i})log(q(x_{i}))$$

wherein, H represents the loss value, p(x) represents the true label value, and q(x) represents the predicted probability.

During the training process, under the constraint of the cross-entropy loss function, the neural network continuously updates its parameters to increase the probability of the model predicting correctly. For instance, if the input spectrogram corresponds to a type of lung cancer, its true label is [0, 1, 0, 0, 0], and the model’s prediction result is, with the cross-entropy loss function yielding a result of. To make the model’s prediction as close as possible to the true result, it is only necessary to minimize the value of the cross-entropy loss function. Therefore, during training, the model will continuously update its parameters to make the output prediction values on the second dimension, corresponding to the true pathological lung cancer, close to 1, whereas the other 4 dimensions approach 0. This enhances the model’s recognition accuracy, achieving the goal of using deep learning algorithms for cough sound recognition.

After the model training is complete, we save the best training weights as the pretrained neural network model. Then, we input the spectrograms from the test set into the pretrained neural network model. In the final layer of the model, the fully connected layer outputs a feature vector, which is then mapped to a set of probability values between 0 and 1 using the Softmax function. These probability values sum up to 1, representing a probability distribution for each possible class. Finally, we select the class with the highest probability as the ultimate prediction.

## Results

### Evaluation Metrics

In this experiment, we used a set of evaluation metrics to assess the performance of the model in the task of cough-related disease classification. These metrics include accuracy, training loss, recall, precision, SD, and *F*_1_-score. We verify the performance of the proposed model by demonstrating a 95% CI for the proposed method [35].

In these evaluations, we used the following terms: true negative (TN), false negative (FN), false positive (FP), and true positive (TP), which represent the following meanings.

For instance, in the case of the COPD category:

- TP: the number of instances correctly classified as COPD, meaning the prediction is COPD, and the ground truth is also COPD.
- FP: the number of instances incorrectly classified as COPD, meaning the prediction is COPD, but the ground truth is non-COPD.
- TN: the number of instances correctly classified as non-COPD, meaning the prediction is non-COPD, and the ground truth is also non-COPD.
- FN: the number of instances incorrectly classified as non-COPD, meaning the prediction is non-COPD, but the ground truth is COPD.
- Accuracy: the proportion of correctly classified samples among all cough samples.
- Precision: the proportion of true positive predictions for the COPD category among all predictions made for the COPD category.
- Recall: the proportion of true positive predictions for the COPD category among all actual COPD samples.
- The SD can be used to measure the range of variation in prediction results. A high SD may indicate that the model is highly sensitive to changes in the input data, leading to greater uncertainty in predictions.
- The CI typically refers to estimating the uncertainty range of a model’s performance metric by using the evaluation results from each fold in the cross-validation process.
- *F*_1_-score: the harmonic mean of precision and recall, resulting in the *F*_1_-score.

Below are the formulas for calculating these evaluation metrics:


(8) $$Accuracy=\frac{TP+TN}{TP+TN+FP+FN}$$

(9) $$Recall=\frac{TP}{TP+FN}$$

(10) $$Precision=\frac{TP}{TP+FP}$$

(11) $$F_{1}\text{-}score=2\times \left(\frac{precision\times recall}{precision+recall}\right)$$

### Model Comparison

To further evaluate the effectiveness of the model, a performance comparison between the proposed CAM-ResNet18 model and the ResNet18 model was conducted on the test dataset. The objective was to observe their precision, recall, *F*_1_-score, SD, positive predictive value, and accuracy. Cross-validation was used to calculate the precision, recall, *F*_1_-score, and their 95% CIs for these 2 models. Specifically, the dataset was divided into 5 folds, and each fold was used for training and validation. The performance metrics for each fold were calculated, and by aggregating the results of all folds, the precision, recall, *F*_1_-score, and their 95% CIs were obtained.

Additionally, we compared the performance of different acoustic features, including spectrogram, MFCC, LPCC, and PLP, when used as input to the CAM-ResNet18 model. This comparison was conducted to identify which feature set yielded the best classification results. We then compared the performance of the CAM-ResNet18 model with common traditional models, such as VGG16, LeNet, and ResNet34. Furthermore, we evaluated the robustness of the model under noise interference by introducing different levels of noise during testing. The results demonstrated how the model’s performance in terms of precision, recall, and *F*_1_-score changed under various noise conditions.

**Figure 6.**
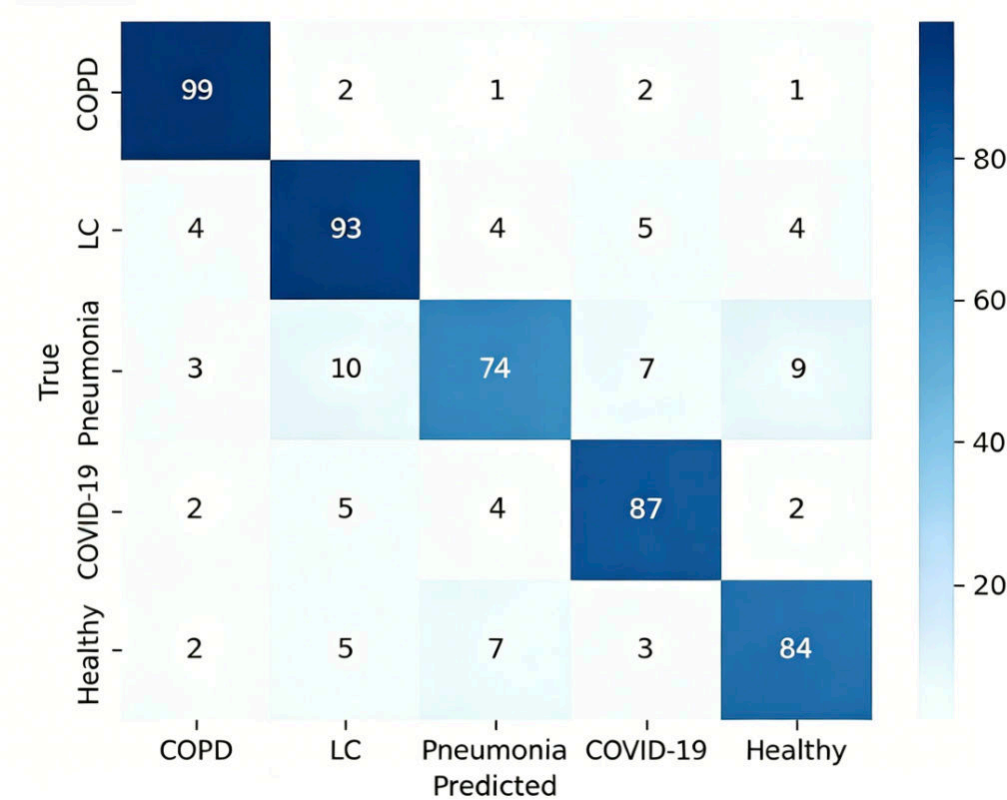
CAM-ResNet18 confusion matrix. COPD: chronic obstructive pulmonary disease; LC: lung cancer.

Finally, we displayed the confusion matrix of the classification results using the CAM-ResNet18 model. These performance metrics are presented in Tables3^8^, as well as Figures6^9^.

**Table 3.** Comparison of different acoustic characteristics of the CAM-ResNet18 model.

Acoustic feature	Precision (%)	Recall (%)	*F*_1_-score (%)
Spectrogram	84.9	87.4	82.52
MFCC^a^	82.1	85.0	80.5
LPCC^b^	80.7	84.3	79.2
PLP^c^	81.3	83.8	79.8

a MFCC: Mel-frequency cepstral coefficient.

b LPCC: LPC cepstral coefficient.

c PLP: perceptual linear prediction.

**Table 4.** Classification performance of the ResNet18 network model.

Performance metric and category	Results (%)	PPV^a^
Precision		
COPD^b^	80.95	73.77‐88.13
Lung cancer	71.42	63.36‐79.48
Pneumonia	75	67.37‐82.63
COVID-19	78.94	71.69‐86.19
Healthy	82.35	75.70‐88.99
Recall		
COPD	85	78.64‐91.36
Lung cancer	83.33	76.60‐90.06
Pneumonia	70.58	62.68‐78.48
COVID-19	78.94	71.69‐86.19
Healthy	77.77	70.37‐85.17
*F*_1_-score		
COPD	82.91	75.97‐89.85
Lung cancer	76.91	75.95‐89.85
Pneumonia	72.72	69.01‐84.81
COVID-19	78.93	71.68‐86.18
Healthy	79.99	72.65‐87.33
Standard deviation		
COPD	1.846	N/A^c^
Lung cancer	2.511	N/A
Pneumonia	2.308	N/A
COVID-19	1.829	N/A
Healthy	2.03	N/A

a PPV: positive predictive value

b COPD: chronic obstructive pulmonary disease.

c N/A: not applicable.

**Table 5.** Classification performance of the CAM-ResNet18 network Model.

Performance metric and category	Results (%)	PPV^a^
Precision		
COPD^b^	90.00	78.28‐93.14
Lung cancer	80.87	69.86‐87.28
Pneumonia	82.22	73.49‐89.01
COVID-19	86.14	76.69‐91.73
Healthy	85.71	81.86‐94.60
Recall		
COPD	94.29	90.52‐98.93
Lung cancer	84.55	78.58‐90.64
Pneumonia	92.36	89.59‐94.85
COVID-19	87.00	82.22‐95.54
Healthy	79.25	71.62‐86.25
*F*_1_-score		
COPD	89.99	84.73‐95.25
Lung cancer	74.59	74.51‐88.43
Pneumonia	76.74	68.78‐84.14
COVID-19	86.54	80.72‐92.24
Healthy	82.38	76.71‐89.93
Standard deviation		
COPD	1.633	N/A^c^
Lung cancer	2.233	N/A
Pneumonia	2.066	N/A
COVID-19	1.6	N/A
Healthy	1.76	N/A

a PPV: positive predictive value.

b COPD: chronic obstructive pulmonary disease.

c N/A: not applicable:

**Table 6.** Comparison of performance across different models.

Model	Accuracy	Specificity	Sensitivity
VGG16	78.6	82.3	80.5
LeNet	79.6	82.9	79.1
ResNet34	82.9	84.3	81.4
CAM-ResNet18	83.9	86.4	87.4

**Table 7.** Classification overall accuracy and average *F*_1_-score of different models.

Network structure	Overall accuracy	Average *F*_1_-score
Resnet-18	78.16	78.29
CAM-Resnet18	83.90	82.52

**Table 8.** Performance table of CAM-ResNet18 model under different noises.

SNR^a^	Precision (%)	Recall (%)	*F*_1_-score (%)
Noiseless	84.9	87.4	82.52
30 dB	83.2	86.1	82.1
20 dB	81.5	84.6	81.0
10 dB	80.6	83.5	79.7

a SNR: signal-to-noise ratio.

**Figure 7.**
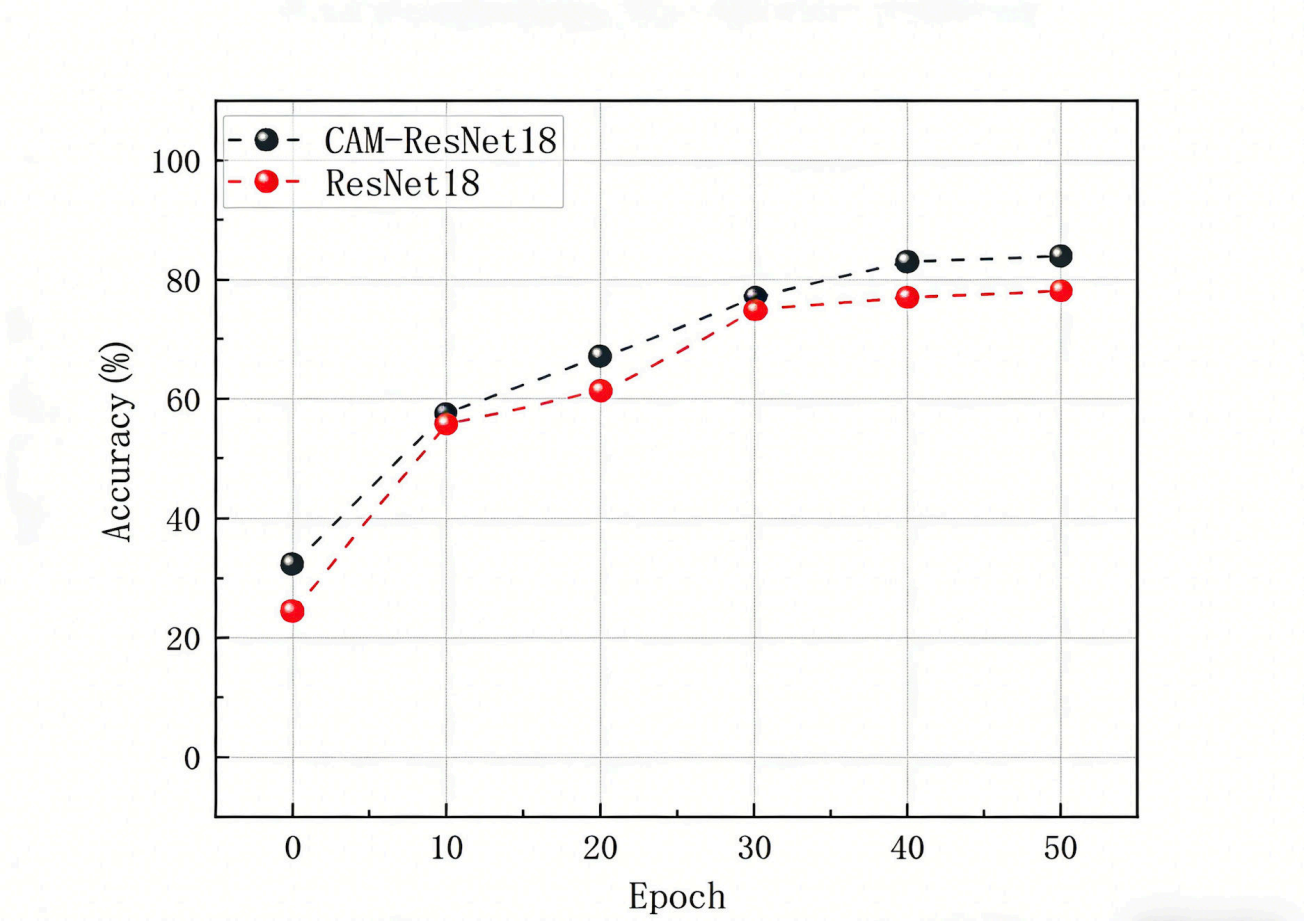
Test the accuracy of CAM-ResNet18 and ResNet models.

**Figure 8.**
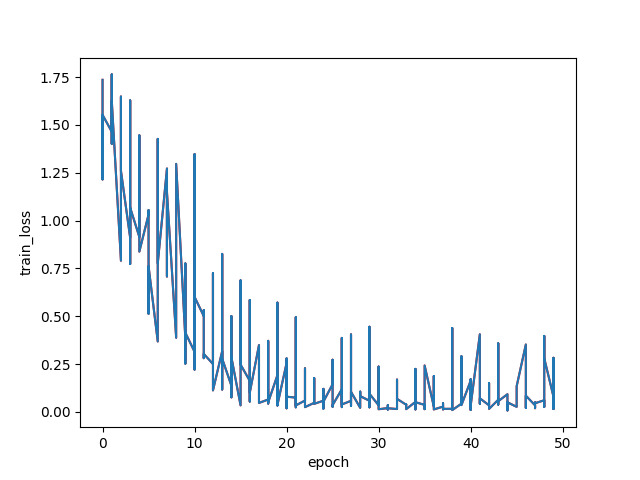
Training loss of CAM-ResNet18.

**Figure 9.**
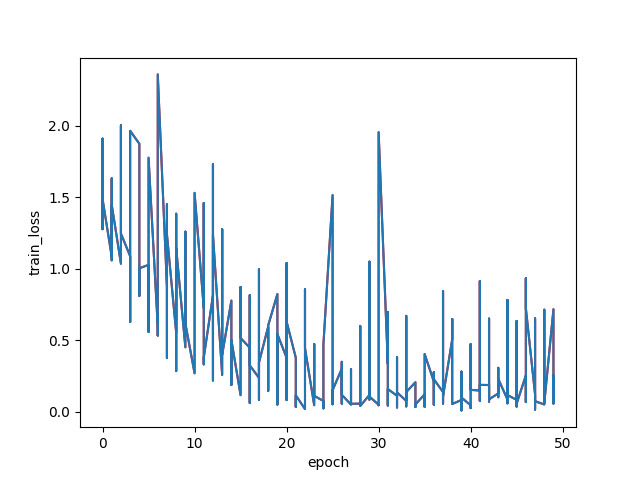
Training loss of ResNet18.

The aforementioned experimental results demonstrate the effectiveness of the CAM-ResNet18 neural network model proposed in this paper. Compared to the traditional ResNet18 network model, the inclusion of the CAM allows the model to focus more on channel features and learn the importance of channels. During the training process, the model’s loss function converges more stably. On the test set, the classification performance of the CAM-ResNet18 network model is significantly better than that of ResNet18, with overall accuracy and average *F*_1_-score improving by 5.74% and 4.23%, respectively, compared to ResNet18. Furthermore, when compared to other models such as VGG16, LeNet, and ResNet34, the proposed CAM-ResNet18 achieved the best performance.

Additionally, through the evaluation of the model’s robustness under noise interference conditions, the model’s performance remained relatively stable at different signal-to-noise ratios, with only a slight decrease in classification accuracy. This indicates that the model possesses certain anti-interference capabilities in real-world application scenarios, allowing it to maintain good classification performance in complex environments. Moreover, we compared different acoustic features, such as MFCC, LPCC, and PLP, and found that the use of spectrograms yielded the best performance across all metrics. This highlights the superior ability of spectrograms to capture relevant audio characteristics for the classification task.

## Discussion

### Principal Findings

To achieve automatic classification and recognition of cough-related diseases, this paper introduces cough classification and audio recognition as a research task. We propose a cough-related disease recognition method that combines an attention mechanism with residual neural networks. We collected cough audio samples related to various diseases (COPD, lung cancer, pneumonia, and COVID-19) and healthy participants to construct a cough audio dataset. All audio samples were preprocessed and transformed into corresponding spectrograms with associated disease labels.

To enhance the model’s focus on key features, improve differentiation among different types of cough sounds, and reduce overfitting without affecting feature extraction in other residual blocks of the neural network, we introduced CAM combined with the ResNet18 neural network. CAM was added to the last convolutional block of each residual block in the ResNet18 network to create a novel neural network model. Because CAM operates independently, it only affects the output feature maps of the current residual block and does not involve the inputs or intermediate feature maps of other residual blocks. It provides local enhancement by weighting the final output feature map to emphasize the importance of specific channels. Furthermore, this unified structure maintains consistency across the entire network and does not disrupt the feature extraction processes of other residual blocks. The cough audio spectrograms from the training set were input into the CAM-ResNet18 network model for training. We used the cross-entropy loss function, Adam optimizer, and adjusted the learning rate to train the model to convergence. Finally, the spectrograms of cough audio from the testing set were input into the converged neural network model, and the Softmax function was used for classification to obtain the final predictions.

### Limitations

This study has several limitations. First, the model used in this study cannot classify cough audio that does not belong to the 5 categories. Although we attempted to classify unknown cough sounds into an “other” category by setting thresholds based on probability values and variance, this method proved to be complex in practice, as determining the appropriate threshold varies across different datasets and application scenarios. Second, the model has not yet achieved high accuracy in classifying cough audio for the 5 categories: COPD, lung cancer, pneumonia, COVID-19, and healthy participants. Manual review and intervention are still required. Third, the cough-related disease classification and identification method proposed in this study has not undergone extensive experimental validation. Therefore, the current approach has certain limitations in practical applications and requires further testing and validation.

Future studies should focus on improving predictive accuracy and enhancing the identification of unknown cough sounds. More complex audio feature parameters such as MFCCs, linear predictive coding coefficients, and speech rate parameters should be considered. Additionally, anomaly detection techniques need to be developed. Furthermore, collecting more samples, expanding the categories for cough-related diseases, and improving cough audio preprocessing are all factors that may contribute to improving the final classification results.

### Conclusions

In the experimental process, we used various performance metrics to evaluate the model’s performance, including accuracy, training loss, recall, precision, and *F*_1_-score. Through comparative experiments with traditional methods, in the audio recognition and classification of healthy participants and the 4 cough-related diseases, COPD, lung cancer, pneumonia, and COVID-19, the proposed CAM-ResNet18 network model showed significantly better classification performance on the collected data compared to traditional methods. The training loss converged more stably during training, and the accuracy and average *F*_1_-score reached 83.9% and 83.54%, respectively, representing an improvement of 5.74% and 4.23% over the ResNet18 network model. The results demonstrate that by introducing the CAM, the model’s focus on specific regions of the spectrogram is enhanced, allowing the model to more effectively explore disease-related features associated with various types of coughing. This is particularly crucial in the field of medicine, as different diseases manifest through distinct sound characteristics. The addition of the CAM to the last convolutional block of each residual block in the ResNet18 neural network effectively captures key features of audio spectrograms related to different types of diseases. Importantly, this addition does not compromise the feature extraction in other residual blocks of the neural network, thereby improving the discriminative power for these 5 types of coughing. Furthermore, in the model training loss plots of the experimental results, we can clearly observe that the training loss of CAM-ResNet18 converges more stably compared to ResNet18. This also validates that the introduction of the CAM improves the convergence performance of the model, enabling it to converge more quickly to the optimal solution, thereby achieving better performance during training. The combined effect of these factors results in superior performance of the CAM-ResNet18 network model in medical image classification tasks.

In addition, compared to current mainstream image classification neural networks, such as LeNet, AlexNet, and VGG16, CAM-ResNet18 has significant advantages in handling audio recognition and classification tasks. First, although LeNet achieved good results in early image classification tasks, its network structure is relatively simple, with fewer layers. Its ability to model complex tasks is limited, making it ineffective at handling high-dimensional and complex input data such as audio. AlexNet, compared to LeNet, made significant improvements by using a deeper network and larger convolutional kernels, which can capture more features. However, when faced with diverse and complex data patterns, AlexNet’s performance is still somewhat limited, especially for audio signals, which have temporal and spectral characteristics, as it lacks sufficient focus on key features.

VGG16 enhances feature extraction by increasing the network depth and using smaller convolutional kernels to capture finer features. However, it has a large number of parameters, high computational complexity, and greater resource demands, which could lead to efficiency issues in practical applications.

In contrast, CAM-ResNet18 significantly improves the extraction of disease-related features from audio signals by introducing the CAM. CAM dynamically adjusts the network’s focus on different regions based on the audio features of various diseases, thus enhancing the model’s discriminative ability. Additionally, ResNet18 avoids the vanishing gradient problem in deep network training through residual connections, allowing for more stable convergence. Compared to VGG16, CAM-ResNet18 has fewer parameters and lower computational complexity, enabling it to maintain high performance even with limited resources.

Our model not only surpasses these traditional image classification networks in audio classification accuracy but also effectively distinguishes between 5 types of audio (healthy, COPD, lung cancer, pneumonia, and COVID-19). As audio signals contain rich temporal and frequency features, CAM-ResNet18 can automatically learn and emphasize disease-related features, making its application in medical diagnostics more widespread and accurate. This ability to distinguish between multiple audio signals makes the model applicable not only to cough recognition but also extendable to more medical audio analysis tasks, such as speech disorder recognition and breath sound recognition, providing strong support for broader health care applications.

Therefore, the method proposed in this study can serve as an adjunct diagnostic tool for clinicians, aiding them in rapidly and accurately identifying the types of diseases in patients during the diagnostic process. This capability is crucial for early detection of diseases, such as COPD, lung cancer, COVID-19, and pneumonia. Early detection can enhance treatment outcomes and mitigate further disease progression. Moreover, automated analysis of cough sound can significantly improve diagnostic efficiency in health care institutions, reducing the workload on health care professionals while enhancing diagnostic objectivity and consistency.

## Abbreviations

CAM: channel attention mechanism
CNN: convolutional neural network
COPD: chronic obstructive pulmonary disease
FN: false negative
FP: false positive
LPCC: linear prediction cepstral coefficient
MFCC: Mel-frequency cepstral coefficient
MLP: multilayer perceptron
PLP: perceptual linear prediction
TN: true negative
TP: true positive

## References

[1] Imran A, Posokhova I, Qureshi HN (2020). AI4COVID-19: AI enabled preliminary diagnosis for COVID-19 from cough samples via an app. Inform Med Unlocked.

[2] Amezquita-Sanchez JP, Mammone N, Morabito FC, Marino S, Adeli H (2019). A novel methodology for automated differential diagnosis of mild cognitive impairment and the Alzheimer’s disease using EEG signals. J Neurosci Methods.

[3] Binnekamp M, van Stralen KJ, den Boer L, van Houten MA (2021). Typical RSV cough: myth or reality? A diagnostic accuracy study. Eur J Pediatr.

[4] Porter P, Brisbane J, Tan J (2021). Diagnostic errors are common in acute pediatric respiratory disease: a prospective, single-blinded multicenter diagnostic accuracy study in Australian emergency departments. Front Pediatr.

[5] Książek W, Abdar M, Acharya UR, Pławiak P (2019). A novel machine learning approach for early detection of hepatocellular carcinoma patients. Cogn Syst Res.

[6] Acharya UR, Oh SL, Hagiwara Y, Tan JH, Adeli H (2018). Deep convolutional neural network for the automated detection and diagnosis of seizure using EEG signals. Comput Biol Med.

[7] Ozturk T, Talo M, Yildirim EA, Baloglu UB, Yildirim O, Acharya UR (2020). Automated detection of COVID-19 cases using deep neural networks with X-ray images. Comput Biol Med.

[8] Eyben F (2015). Real-Time Speech and Music Classification by Large Audio Feature Space Extraction.

[9] Yang J (2011). Combining speech enhancement and cepstral mean normalization for LPC cepstral coefficients. Key Eng Mater.

[10] Dubey RK, Kumar A Non-intrusive objective speech quality assessment using a combination of MFCC, PLP and LSF features.

[11] Sreeram ASK, Ravishankar U, Sripada NR, Mamidgi B Investigating the potential of MFCC features in classifying respiratory diseases.

[12] Chambres G, Hanna P, Desainte-Catherine M Automatic detection of patient with respiratory diseases using lung sound analysis.

[13] Aykanat M, Kılıç Ö, Kurt B, Saryal S (2017). Classification of lung sounds using convolutional neural networks. J Image Video Proc.

[14] Yue L, Xu W Automatic classification of childhood asthma and pneumonia based on cough sound analysis.

[15] Pahar M, Klopper M, Warren R, Niesler T (2021). COVID-19 cough classification using machine learning and global smartphone recordings. Comput Biol Med.

[16] Mouawad P, Dubnov T, Dubnov S (2021). Robust detection of COVID-19 in cough sounds. SN COMPUT SCI.

[17] Huang YP, Mushi R (2022). Classification of cough sounds using spectrogram methods and a parallel-stream one-dimensional deep convolutional neural network. IEEE Access.

[18] Bales C, Nabeel M, John CN Can machine learning be used to recognize and diagnose coughs?.

[19] Bansal V, Pahwa G, Kannan N Cough classification for COVID-19 based on audio MFCC features using convolutional neural networks.

[20] Morice AH (2006). Chronic cough: diagnosis, treatment and psychological consequences. Breathe (Sheff).

[21] He K, Zhang X, Ren S, Sun J Deep residual learning for image recognition.

[22] Krizhevsky A, Sutskever I, Hinton GE (2017). ImageNet classification with deep convolutional neural networks. Commun ACM.

[23] Sharan RV, Abeyratne UR, Swarnkar VR, Porter P (2019). Automatic croup diagnosis using cough sound recognition. IEEE Trans Biomed Eng.

[24] Porter P, Abeyratne U, Swarnkar V (2019). A prospective multicentre study testing the diagnostic accuracy of an automated cough sound centred analytic system for the identification of common respiratory disorders in children. Respir Res.

[25] Chatrzarrin H, Arcelus A, Goubran R, Knoefel F Feature extraction for the differentiation of dry and wet cough sounds.

[26] Loey M, Mirjalili S (2021). COVID-19 cough sound symptoms classification from scalogram image representation using deep learning models. Comput Biol Med.

[27] Rocha BM, Pessoa D, Marques A, Carvalho P, Paiva RP Personalized detection of explosive cough events in patients with pulmonary disease.

[28] Kaplan A, Cao H, FitzGerald JM (2021). Artificial intelligence/machine learning in respiratory medicine and potential role in asthma and COPD diagnosis. J Allergy Clin Immunol Pract.

[29] Orlandic L, Teijeiro T, Atienza D (2021). The COUGHVID crowdsourcing dataset, a corpus for the study of large-scale cough analysis algorithms. Sci Data.

[30] Fakhry A, Jiang X, Xiao J, Chaudhari G, Han A, Khanzada A (2021). Virufy: a multi-branch deep learning network for automated detection of COVID-19. arXiv.

[31] Zhang Z, Wang M (2022). Convolutional neural network with convolutional block attention module for finger vein recognition. arXiv.

[32] Taud H, Mas J (2018). Multilayer Perceptron (MLP).

[33] Hu K, Zhang Z, Niu X (2018). Retinal vessel segmentation of color fundus images using multiscale convolutional neural network with an improved cross-entropy loss function. Neurocomputing.

[34] Altan G (2022). DeepOCT: an explainable deep learning architecture to analyze macular edema on OCT images. Eng Sci Technol Int J.

[35] Altan G, Kutlu Y, Allahverdi N (2020). Deep learning on computerized analysis of chronic obstructive pulmonary disease. IEEE J Biomed Health Inform.

